# PCA-Based Hybrid Intelligence Models for Estimating the Ultimate Bearing Capacity of Axially Loaded Concrete-Filled Steel Tubes

**DOI:** 10.3390/ma15186477

**Published:** 2022-09-18

**Authors:** Kaffayatullah Khan, Rahul Biswas, Jitendra Gudainiyan, Muhammad Nasir Amin, Hisham Jahangir Qureshi, Abdullah Mohammad Abu Arab, Mudassir Iqbal

**Affiliations:** 1 Department of Civil and Environmental Engineering, College of Engineering, King Faisal University, Al-Ahsa 31982, Saudi Arabia; 2 Department of Applied Mechanics, Visvesvaraya National Institute of Technology, Nagpur 440010, India; 3 Department of Civil Engineering, GLA University, Mathura 281406, India; 4 Department of Civil Engineering, University of Engineering and Technology, Peshawar 25120, Pakistan

**Keywords:** structural analysis, thin-walled structure, artificial neural network, principal component analysis, dimension reduction, accuracy matrix

## Abstract

In order to forecast the axial load-carrying capacity of concrete-filled steel tubular (CFST) columns using principal component analysis (PCA), this work compares hybrid models of artificial neural networks (ANNs) and meta-heuristic optimization algorithms (MOAs). In order to create hybrid ANN models, a dataset of 149 experimental tests was initially gathered from the accessible literature. Eight PCA-based hybrid ANNs were created using eight MOAs, including artificial bee colony, ant lion optimization, biogeography-based optimization, differential evolution, genetic algorithm, grey wolf optimizer, moth flame optimization and particle swarm optimization. The created ANNs’ performance was then assessed. With R^2^ ranges between 0.7094 and 0.9667 in the training phase and between 0.6883 and 0.9634 in the testing phase, we discovered that the accuracy of the built hybrid models was good. Based on the outcomes of the experiments, the generated ANN-GWO (hybrid model of ANN and grey wolf optimizer) produced the most accurate predictions in the training and testing phases, respectively, with R^2^ = 0.9667 and 0.9634. The created ANN-GWO may be utilised as a substitute tool to estimate the load-carrying capacity of CFST columns in civil engineering projects according to the experimental findings.

## 1. Introduction

In recent times, high-rise and large-scale building structures have become more popular in demand, and the use of concrete-filled steel tube (CFST) columns in those structures has increased due to the ductility and energy absorption capacity, which is significantly more compared to the conventional reinforced concrete (RCC) members. CFST is a composite member, made up of steel with concrete. 

Hence, the main advantage of CFST is to make use of both type of materials, which not only enhances the toughness and plasticity of concrete but also delays the local buckling of tabular steel. Due to the exceptional static and dynamic (earthquake-resistant) characteristics of CFST columns, they are also used in earthquake-resistant structures, bridge piers (which are subjected to traffic), in railway decks and as pile in high-rise buildings [1].

In CFST columns, the main structural advantage is due to the confinement effect of the steel, which surrounds the concrete, and also due to the contribution of steel to the load-carrying capacity. However, the time consumed for construction is also reduced because of the elimination of a permanent formwork. The delay in the local buckling of steel due the concrete core is also one of the major benefits of using CFST columns [2,3]. Hence, from past studies [2,3,4,5,6,7,8], it can be concluded that the use of CFST columns increases the load-carrying capacity, ductility and stiffness and is economical and less time consuming in construction, which makes CFST columns an attractive solution in the field of civil engineering [9,10].

Tests for CFST filled with high strength concrete and of different cross-sections (i.e., circular, rectangular, square and elliptical) have been reported [1,11,12,13,14,15,16,17,18,19,20,21]. Giakoumelis et al. [22] performed a study to examine the effect of several factors on CFSTs with different concrete strengths under the axial load. In another study, Evirgen et al. [23] studied 48 CFSTs under axial compression and explored the effects of the geometrical shape of specimens, concrete strength and width/thickness ratio on ultimate loads. The behaviour of CFST columns was analysed through experimental studies on twenty-six samples subjected to axial compressive loading, with different strengths of concrete, by Jamaluddin et al. [24]. 

These studies have shown that global and local buckling characterizes the failure of stub and slender CFST. It was also observed in the study that the high strength concrete improves the ductility of CFST over normal concrete. In addition, the axial performance also relies on the slenderness of the steel tube, which was checked by Lam et al. [25] who found that the load-bearing capacity of the CFST columns reduces by increasing the tube thickness. Numerical approaches, such as the finite element method, have been used to research the structural efficiency of compressive CFST members to decrease the expense of experimentation. 

Lui et al. [26] proposed a numerical simulation technique to predict the ultimate load, which was found to be efficient and less time consuming. In other studies, both Hans et al. and Tao et al. developed a finite element model by considering interaction and nonlinearity between the steel and concrete and validated the model satisfactorily with previous works. Whereas, an ABAQUS simulation was performed by Lyu et al. [27] to analyse the failure mode and ultimate bearing capacity of square CFST columns with reinforcement stiffener at different temperatures. The ultimate axial capacity is an important index to assess the applicability of CFST columns under axial compression in both numerical simulation and laboratory experiments [13,28,29,30,31,32,33].

Several codified formulations have been implemented at the same time to estimate the potential of CFST columns in compression, including the Standards American Institute of Steel Construction [34], Standards Association of Australia [35], Architectural Institute of Japan [36], Chinese code DL/T [25] and European Committee for Standardization Eurocode 4 [37]. Many empirical formulas have also been proposed in previous studies, including Sakino et al. [38], Han and Yao [39], Lu and Zhao [2] and Hatzigeorgiou [40]. 

The results found using the CISC formula are extremely underestimated with the experimental results, along with the other codes, such as AS4100, AS3600, AIJ-1997 and ACI-318R, whereas the models proposed by Lam, Hatzigeorgiou [22,40] and Lu and Zhao [2] underestimated the maximum results. In brief, the laboratory tests of these compression tests are laborious and time consuming and the numerical simulation is also difficult due to the material properties and complicated conditions. Hence, the researchers adopted alternative soft computing techniques to conveniently evaluate the accurate axial ultimate compression values [41,42,43,44,45,46,47,48,49,50,51,52,53].

Artificial intelligence (AI) approaches have been successfully employed in diverse areas in the last few decades [54,55,56,57,58,59,60,61]. Many experiments concerning artificial intelligence have been performed in terms of CFST columns in order to study their behaviour under different forms of loading. For example, the output of circular CFST subjected to axial compressive load was investigated in Kheyroddin et al. [62] and Guneyisi et al. [14] using ANN and gene expression programming, respectively. Apart from this, the ANN technique was also implemented in rectangular CFST columns to find the bearing capacity of the same by Du et al. [63] and Sarir et al. [64]. 

To predict the CFST load-carrying potential in the prediction and optimization stages, several advanced techniques were developed. As seen in the literature, the results obtained indicate that AI techniques give promising prospects for predicting the mechanical behaviour of structural components. While different AI strategies have been used to predict CFST’s mechanical responses, other interesting methods may be used improve the prediction efficiency—for example, the hybrid ANN models [65,66,67,68]. Few studies have explored the feasibility of using hybrid models of ANN in terms of forecasting CFST’s load-carrying capacity.

## 2. Research Significance

Currently, the behaviour of CFST columns under axial load is an important aspect of study due to its high efficiency than normal concrete columns. As the CFST members are a complex system, their strength properties depend on the material constituents and the involved construction techniques and parameters. Though there have been numerous studies regarding the prediction of the axial load on CFST members, it still remains an issue with substantial attention in structural engineering, and is also mentioned in ACI-318R [69], AS4100 [70], AIJ-1997 [36], AISC [34], Eurocode 4 [71], Giakoumelis and Lam [22] and Hatzigeorgiou [40]. 

This is driven by the fact that, under axial compression, the mechanical behaviour of CFST exhibits a strong nonlinear nature extracted from the mechanical and geometric factors involved in their behaviour. In this research, hybrid ANN-based models with and without principal component analysis (PCA) were used to predict the load-carrying capacity of CFST under uniaxial compression as they are effective in exploring the complicated and nonlinear relationship of the data. This study is aimed to develop the models, which will be more effective in overcoming expensive and time-consuming experiments. 

The following points constitute the contributions of the present work: (a) the development of eight hybrid ANN-based algorithms with the dimension reduction technique (i.e., principal component analysis (PCA) and employed meta-heuristic algorithms—namely, artificial bee colony (ABC), ant lion optimization (ALO), biogeography-based optimization (BBO), differential evolution (DE), genetic algorithm (GA), grey wolf optimizer (GWO), moth flame optimization (MFO) and particle swarm optimization (PSO)) for forecasting the CFST’s load-carrying capacity under uniaxial compression; (b) the optimization procedure of models ANN-ABC, ANN-ALO, ANN-BBO, ANN-DE, ANN-GA, ANN-GWO, ANN-MFO and ANN-PSO are used along with the verification process to confirm that no overestimation occurrs; (c) within a convergent, probabilistic context, uncertainty analysis and robustness over 149 sample results in total are performed; and (d) from a physical point of view, the effect of input variables on the prediction of column load-carrying capacity is investigated.

## 3. Methodology

### 3.1. Principal Component Analysis

PCA is a reputed and prominent method of data reduction and feature extraction. The fundamental property of PCA is to find a smaller set of uncorrelated components from a significantly bigger predictor variable (high dimensional inputs) by computing Eigen vectors from covariance matrix. For the mathematical formulation, a set of m predictor variables can be denoted by:

(1) $$u_{i}={\left(u_{i}\left(1\right),u_{i}\left(2\right),\ldots ,u_{i}\left(p\right)\right)}^{T};\;i=1,2,...,q.$$

The sample covariance matrix is given by:

(2) $$M=\frac{1}{q}\overset{q}{\underset{i=1}{\sum }}u_{i}.u_{i}{}^{T}$$

In PCA, predictor variables are transformed to new variables as:

(3) $$v_{i}=U^{T}u_{i},$$

where U is p×p orthogonal matrix. The *j*th Eigen vector of the sample covariance matrix corresponds to the the *j*th column ($C_{j}$) of the U matrix. The following expression is used to solve it.

(4) $$\lambda_{j}r_{j}=Mr_{j},j=1,\;2,\ldots ,p$$

where $\lambda_{j}$ and $r_{j}$ represent the Eigen value and corresponding Eigen vector of M, respectively. Upon transformation of $u_{i}$, the orthogonal portion of predictor variable vi is calculated using Equation (1). The resulting component is recognized as the principal component. The predictor variable is reduced to principal components whose selection is the function of Eigen values post arranging Eigen vectors in descending order. Thus, the dimension of predictor variables is reduced to principal components in PCA. They are uncorrelated and have maximum variances sequentially.

### 3.2. Artificial Neural Network

ANN is an artificial computational system made up of artificial neurons that mimic the parallel processes of a biological brain in order to find the answer. It is made up of artificial neurons that play the role of fundamental units and mimic the organisational principles of the human nervous system. Due to its capacity to learn automatically from provided training patterns, ANN addresses the mapping problem by identifying the closest association between the input and output parameter [72,73]. In more technical terms, the network’s architecture and connection weights change repeatedly until the error at each output layer node is minimised. *E*, a squared error function, calculates the output error as follows:

(5) $$E=\frac{1}{2}\overset{P}{\underset{i=1}{\sum }}{\left(t^{\left(i\right)}-y^{\left(i\right)}\right)}^{2}$$

where *t* is the target value, *y* is the actual value and *P* stands for the number of training patterns. Back-propagation (BP) learning is a gradient-based learning process that is commonly employed for network learning tasks [65,74]. Any training session in the BP learning algorithm is a twofold approach that comprises both forward and backward stages. In the forward stage, input signals go through the network, and each output layer node emits an error signal. Then, in the next phase [75], the rates of the resultant error traverse backward along the network, correcting the network’s weights and biases. 

**Figure 1:**
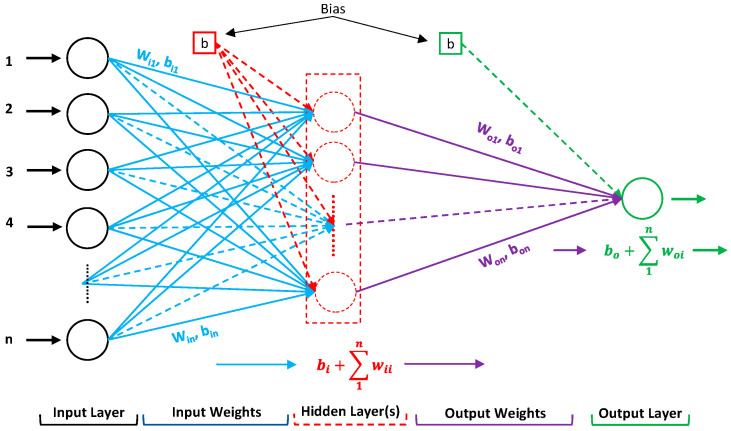
A typical layout of ANN with a single hidden layer.

The multilayer perceptron (MLP) neural network is one of the most used approaches for developing an ANN model, since it can handle complicated mathematical problems that involve nonlinear equations by establishing correct weights. At least three layers contribute to a typical MLP. The first layer is referred to as the input layer, the last layer is referred to as the output layer, and the levels in between are referred to as hidden layers. A typical illustration of an ANN architecture is shown in Figure 1.

### 3.3. Overview of Employed MOAs

Meta-heuristic approaches are explored in this section. In general, the usage of meta-heuristics optimization algorithms (MOAs) in the field of engineering to solve various problems has increased significantly. These are free gradient methods that may tackle extremely difficult optimization problems with better outcomes compared with standard approaches [76]. Furthermore, they are easier to build and faster than traditional optimization approaches [77]. There are several sources of inspiration for MOAs, which may be categorised into distinct groups based on these sources of inspiration. Evolutionary algorithms (EAs), swarm intelligence (SI) methods, natural phenomena approaches and human-inspiration algorithms are among these categories. 

**Figure 2:**
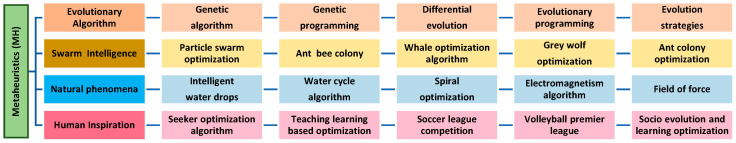
The different categories of MOAs.

Figure 2 displays these groupings. The motivation for the algorithms in the first category, known as EAs, comes from simulating natural genetic processes, such as crossover, mutation and selection. Evolutionary programming, evolutionary strategy (ES), GA, DE and genetic programming (GP) are some of the MOAs that fall within this category. The second group, called SI, replicates swarm behaviours in nature when looking for food. The PSO, ABC, GWO, ACO, salp swarm algorithm (SSA), marine predators’ algorithm (MPA) and whale optimization algorithm (WOA) are the most prominent members of this category. 

The third group attempts to imitate natural phenomena, such as rain, spirals, wind and light. The water cycle algorithm (WCA), spiral optimization (SO), intelligent water drops (IWD), electromagnetism algorithm and field of force (FF) all members of this category. Furthermore, additional procedures fall under this category but are based on physical rules—for instance, electromagnetism algorithm, field of force (FOF), charged system search (CSS), simulated annealing, gravitational search algorithm (GSA), aquila optimizer (AO), flow regime algorithm (FRA), electromagnetism-like mechanism, charged system search (CSS), chemical-reaction-inspired meta-heuristic and optics-inspired optimization (OIO). 

In addition, the fourth category is influenced by human activities: volleyball premier league algorithm (VPL), teaching learning-based optimization (TLBO), soccer league competition (SLC), league championship algorithm (LCA), seeker optimization algorithm (SOA) and socio evolution and learning optimisation (SELO) are examples of algorithms in this category.

### 3.4. Construction Procedure of Hybrid ANNs

MOAs are used to improve the performance of conventional machine learning (CML) approaches by optimising their learning parameters (such as the weights and biases). By refining the learning parameters of CML approaches, the integration of CML and MOA aids in the search for the precise global minimum, resulting in more accurate outcomes [66,78,79,80,81,82,83]. To maximise the learning parameters of ANN, advanced MOAs (ABC, ALO, BBO, DE, GA, GWO, MFO and PSO) were employed to develop hybrid ANN models in this work. Input weights, hidden biases, output weights and output biases are the learning parameters of an ANN. 

The methodological evolution of ANN-based hybrid models may be summarised as follows: In the first step, hyper-parameters (such as *N_hn_* and the activation function) are chosen, and weights and biases are generated at random followed by the development of optimum learning parameter values using MOAs in the second stage. Finally, utilising the adjusted weights and biases, the developed hybrid ANN models are used for the new dataset to validate the results. While the methodology for creating hybrid models is the same for every MOA, the developed optimum learning parameters are not the same. 

In addition to the ANN’s learning parameters, deterministic parameters, such as the population size (*N_p_*), generation probability (*GP*), maximum number of iterations (*itr*), inertia weights (*w_max_* and *w_min_*), random parameters (*r*_1_, *r*_2_), acceleration coefficients of PSO (*c*_1_ and *c*_2_), lower bound (*lb*), upper bound (*ub*) and other MOA parameters, are important and, therefore, should be tuned appropriately during hybrid modelling. 

## 4. Data Processing and Analysis

### 4.1. Descriptive Statistics and Statistical Analysis

As mentioned above, a sum of 149 experimental results for stub/short CFSTCs were collected from 22 different sources mentioned in the paper of Cigdem Avci-Karatas [12] and will be used to develop a hybridised ANN model with the dimension reduction technique (i.e., PCA) and a Convolutional Neural Network (CNN). 

The input parameters for the study are the wall thickness of the steel tube (*D*), outer diameter of the steel tube (𝐷), unconfined concrete strength (𝑓_𝑐_), yield strength of the steel (𝑓_𝑦_), Young’s modulus of concrete (𝐸_𝑐_), Young’s modulus of the steel (𝐸_𝑠_) confinement factor (𝜉) and length of CFSTC (*L*), whereas the effects of d/t and l/d were also considered on the CFST’s load-carrying capacity under uniaxial compression.

Table 1 shows the descriptive statistic of the input and output parameters where it can be seen that the D varies from 60 to 450, t varies from 0.86 to 10.36, 𝑓_𝑐_ and 𝑓*_y_* vary from 18.03 to 853, *E_c_* and *E_s_* vary from 17,810 to 213,000 and the output value *P_u_* varies from 312 to 13,776 indicating the wide variety of experimental data. Statistical analysis was undertaken in order to measure the degree of correlation (DOC) between the above parameters after the descriptive analysis described above revealed that the collected database had a wide range of experimental data. 

**Figure 3:**
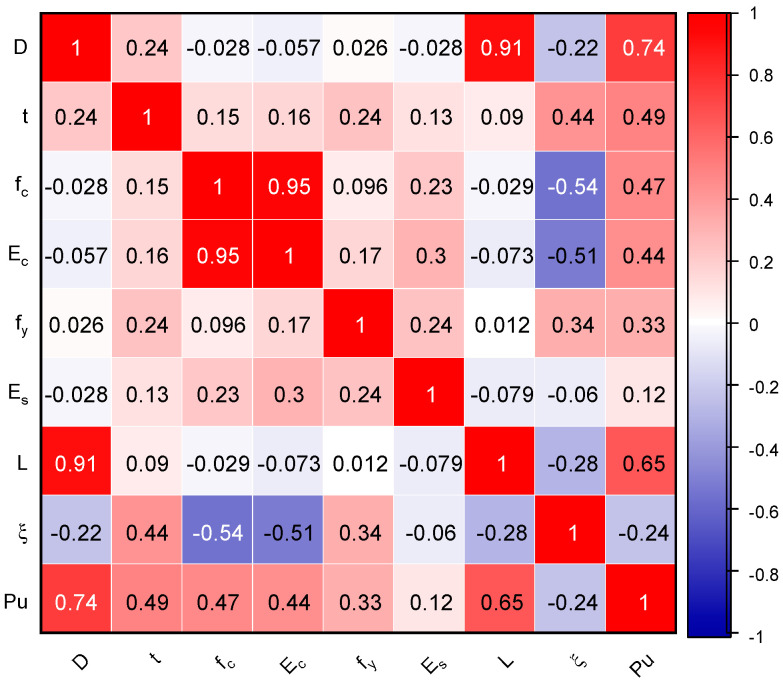
Correlation matrix.

**Figure 4:**
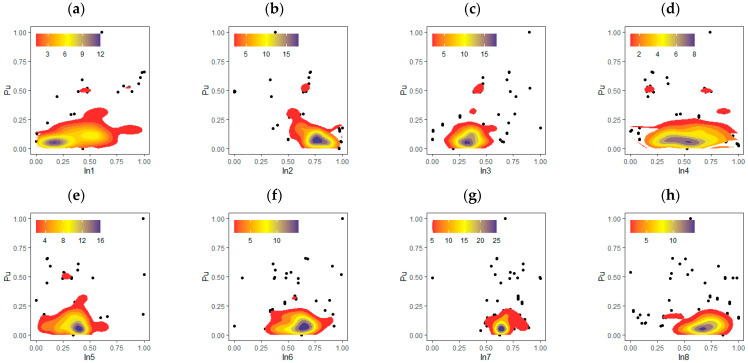
Scatter plot with 2-D density estimation between inputs parameters (**a**–**h**) and P_u_.

When all parameters are evaluated, the DOC between *P_u_* and other parameters (excluding D, L, t and *f_c_*) is smaller, according to the information provided by the Pearson correlation in Figure 3. The DOC between *P_u_* with D, L, t and *f_c_* on the other hand, was shown to be significantly higher. However, a closer look indicates that the experimental dataset had a large number of uncorrelated data points. The collected dataset is also displayed in Figure 4 as a scatterplot with 2-D density estimation between inputs and output variables. This helps to visualise the nature of the input characteristics.

### 4.2. Data Processing Using PCA

The DOC between the attributes (CFST parameters) was detected on the higher side in certain cases in the experimental database, while it was observed on the lower side in many situations, indicating multicollinearity among the variables. Furthermore, the experimental database had eight attributes and 150 observations, resulting in a high-dimensional dataset. As a result, PCA was used in this study to eliminate the multicollinearity and dimensionality effects. The number of input variables was then chosen based on the entropy idea, which explains the greatest amount of variance in the dataset. 

All of the new variables are orthogonal to one another, which solves the multicollinearity and dimensionality difficulties. PCA produces an equal number of PCs in most cases; however, the ideal number may be determined using the cumulative percentage of variance (CPOV). In Table 2, the PCA realisations, including the proportion of variance (POV), standard deviation (SD) and CPOV of PCs, are presented. 

**Figure 5:**
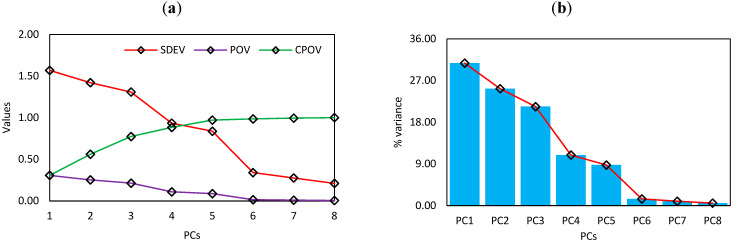
(**a**) Plot of SD, POV and CPOV of PCA and (**b**) scree plot.

**Figure 6:**
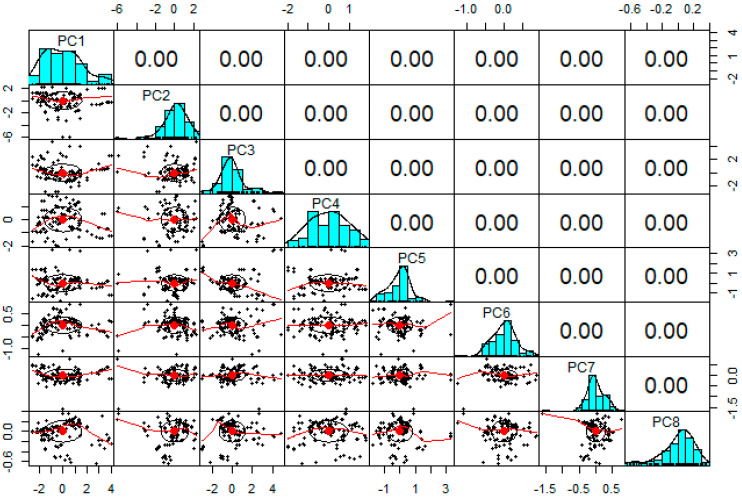
Pair plot of PCs.

The rotations of PCs are presented in Table 3. The percentage of explained variance and scree plot are presented Figure 5 and Figure 6, respectively. From the information presented in Table 2, it can be seen that PC1 to PC8 cover 100% of total variance. This suggests that all of the elements have a role and are likely to be significant. The PC chosen is based on the researcher’s preferences and the type of problem. However, the pair plots of PCs are presented in Figure 6.

### 4.3. AI-Based Analysis

Data normalisation is a pre-processing task in the field of machine learning that is usually performed to eliminate multi-dimensional effects. As a result, the number of input variables was chosen based on the cumulative variance of shortly after PCA was implemented to deal with multicollinearity issues. Using the normalisation procedure, the new dataset with eight inputs and 149 observations was normalised between 0 and 1. Following that, the normalised dataset was split into two parts: training and testing. 

**Figure 7:**
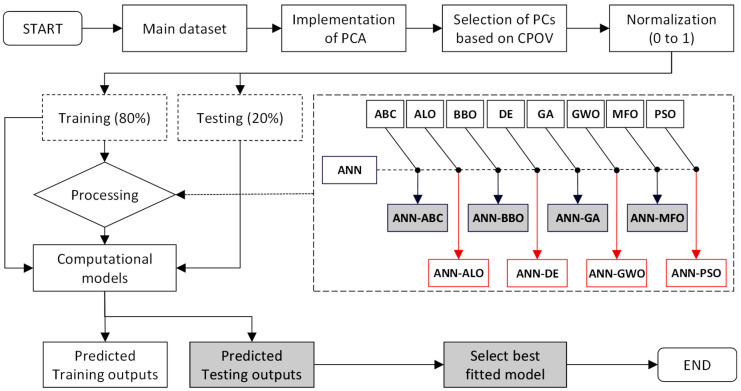
Steps showing the implementation of PCA-based hybrid ANNs.

The training dataset was chosen at random from the main dataset, and the testing dataset was chosen from the remaining dataset. The researchers must assess the prediction models’ performance because there are no criteria for splitting the dataset into training and testing groups. A tiny number of testing datasets for assessing the prediction models’ performance, on the other hand, cannot be regarded as important. Figure 7 depicts the full hybridization process, including the procedures for building the ANN-based hybrid models ANN-ABC, ANN-ALO, ANN-BBO, ANN-DE, ANN-GA, ANN-GWO, ANN-MFO and ANN-PSO.

To evaluate the performance of the developed models, eight different performance indices, such as the determination coefficient (R^2^), Willmott’s index of agreement (WI), the Nash–Sutcliffe efficiency (NSE), performance index (PI), root mean square error (RMSE), mean absolute error (MAE), mean absolute percentage error (MAPE) and weighted mean absolute percentage error (WMAPE), were determined [66,67,84,85,86,87,88,89,90,91,92,93,94,95,96,97,98,99,100,101,102,103,104,105,106]. Note that, for a perfect predictive model, the values of these indices should be equal to their ideal value given in Table 4. It may also be noted that these parameters are usually determined to assess the generalization capability of any predictive models from different aspects, such as the degree of correlation, associated error and amount variances.

(6) $$R^{2}=\frac{\sum_{i=1}^{n}{\left(y_{i}-y_{mean}\right)}^{2}-\sum_{i=1}^{n}{\left(y_{i}-{\hat{y}}_{i}\right)}^{2}}{\sum_{i=1}^{n}{\left(y_{i}-y_{mean}\right)}^{2}}$$

(7) $$WI=1-\left[\frac{\sum_{i=1}^{n}{\left(y_{i}-{\hat{y}}_{i}\right)}^{2}}{\sum_{i=1}^{n}{\left\{\left|{\hat{y}}_{i}-y_{mean}\right|+\left|y_{i}-y_{mean}\right|\;\right\}}^{2}}\right]$$

(8) $$NSE=1-\frac{\sum_{i=1}^{n}{\left(y_{i}-{\hat{y}}_{i}\right)}^{2}}{\sum_{i=1}^{n}{\left(y_{i}-y_{mean}\right)}^{2}}$$

(9) $$PI=adj.R^{2}+0.01VAF-RMSE$$

(10) $$RMSE=\sqrt{\frac{1}{n}\overset{n}{\underset{i=1}{\sum }}{\left(y_{i}-{\hat{y}}_{i}\right)}^{2}}$$

(11) $$MAE=\frac{1}{n}\overset{n}{\underset{i=1}{\sum }}\left|\left({\hat{y}}_{i}-y_{i}\right)\right|$$

(12) $$MAPE=\frac{1}{n}\overset{n}{\underset{i=1}{\sum }}\left|\frac{y_{i}-{\hat{y}}_{i}}{y_{i}}\right|\times 100$$

(13) $$WMAPE=\frac{\sum_{i=1}^{n}\left|\frac{y_{i}-{\hat{y}}_{i}}{y_{i}}\right|\times y_{i}}{\sum_{i=1}^{n}y_{i}}$$

where $y_{i}\;$and ${\hat{y}}_{i}\;$are the actual and estimated *i*th value; *n* and *P* are the number of samples and number of input parameters in a dataset under consideration; and $y_{mean}$ is the average of the actual values.

## 5. Results and Discussion

### 5.1. Parametric Configuration

In the below sub section, the results of the hybrid ANN using PCA for estimating the ultimate load-carrying capacity of CFST are presented. As discussed above, eight PCs (PC1 to PC8) were selected based on PCA to predict the ultimate load-carrying capacity. The dataset was divided into training and testing sets where the model was developed with the help of the training dataset, while the testing dataset was used to validate the model. Hence, the performance of developed model was evaluated using various indices for both training and testing.

However, before analysing the results, it is important to discuss the configuration of different hyper and deterministic parameters as presented in Table 5. It is evident that the deterministic parameters play an important role to develop a model. Hence, the hyper-parameters of ANN, such as $N_{hn}$. and the activation functions, were tuned properly. In order to find the optimum value of $N_{hn}$, the parameter was tuned in the range of 5 to 25, and we found that the optimum value of $N_{hn}$ was 10. In this study, the sigmoid function was used as the activation function. 

The deterministic parameters of metaheuristic optimization algorithms (MOA), such as $N_{p}$, $w_{\max}$**,** $w_{\min}$, itr, c_1_ and c_2_, were also tuned during the simulation. The optimum values of the effective parameters influencing the developed models are shown in Table 5. It can be seen from Table 5 that, for fair comparison, the values of $N_{hn}$, $N_{hl}$ and $N_{p}$ were kept constant for all the hybrid ANN models. Therefore, the total number of optimized learning parameters $\left(N_{lp}\right)$ of the ANN-based model was 101 ($N_{hn}$ × number of input neurons+ number of hidden biases + hidden to output weight + output bias, i.e., 8 × 10 + 10 + 10 + 1).

In the case of ANN-based modelling, the ANN model was initialized first, and after that, the MOAs (ABC, ALO, BBO, DE, GA, GWO, MFO and PSO) was applied with PCA to optimize the learning parameters of ANN through the cost function, RMSE. In order to find the optimum value of learning parameter, 500 iterations were performed with varying the $N_{hn}=5\;\mathrm{to}\;25$ and $N_{p}=50$. The MOAs were tuned using trial and error process to obtain the best possible values of the output. Those values were used to optimize the weights and biases of the hybrid ANN models. Further, it should be noted that the convergence behaviours during the iterative process of MOAs is one of the major factors to access the performance of models. The convergence behaviour illustrates the capability of MOAs to ignore the local minima. 

**Figure 8:**
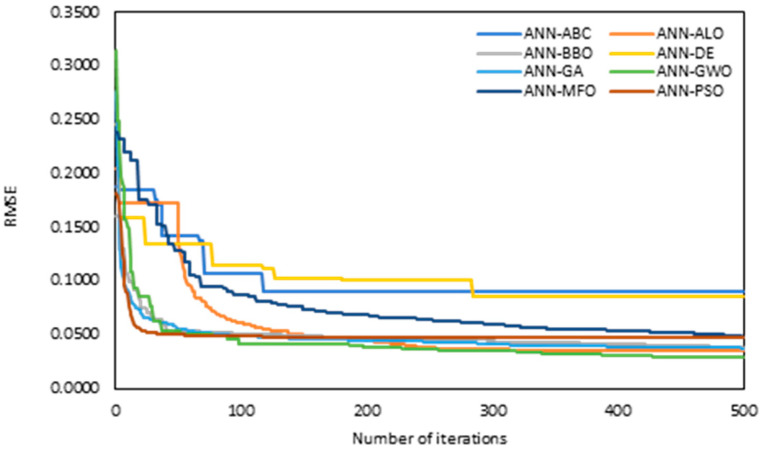
Convergence curves of PCA-based hybrid ANNs.

The convergence behaviour of hybrid ANN with PCA is shown in Figure 8. From the figure, it is evident that ANN-GWO achieved the best convergence followed by ANN-PSO, ANN-BBO and ANN-GA, whereas the ANN-DE and ANN-ABC had the worst convergence.

### 5.2. Model Performance

The prediction capability of the proposed hybrid ANN models with PCA were investigated to predict the load-carrying capacity of CFST columns. In this section, the comparative analysis of statistical parameters for quality assessment for both training and testing parameters is presented in Table 6 and Table 7, respectively. The summary of score analysis is highlighted in Table 8. For the purpose of characterizing the robustness of the AI models, the R^2^, RMSE, WI, NSE, PI. NS, RSR, MAE and WMAPE were calculated and are presented in the tables. The main objective of the study was to perform a comparative analysis of the hybrid ANN models.

The performance indices and score analysis for the training and testing phase of the hybrid ANN models are tabulated in Table 6 and Table 7. For the training phase, the ANN-GWO yielded the highest value with the score of 64 and R^2^ = 0.9667, whereas the ANN-ABC and ANN-DE underperformed among all the models with the overall scores of 8 and 16, respectively. However, the results of ANN-ALO, ANN-GA and ANN-BBO were good in the training phase with R^2^ values of 0.9527, 0.9463 and 0.9460, respectively. 

**Figure 9:**
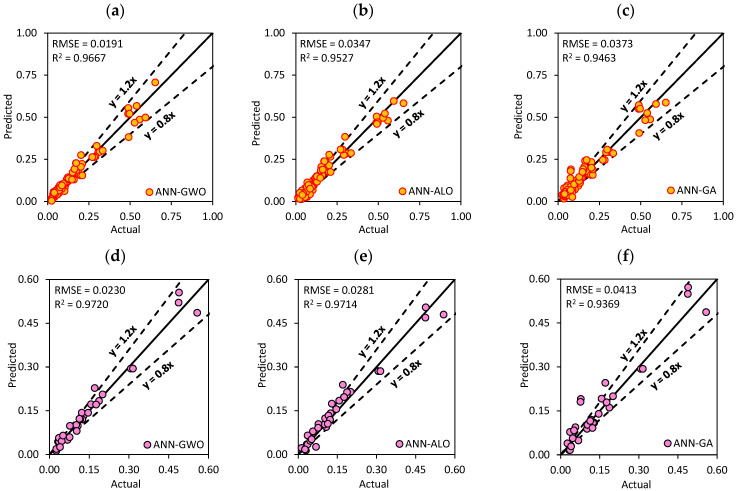
Scatter plots between the actual and predicted values for the best three models: (**a**–**c**) training and (**d**–**f**) validation datasets.

Figure 9a–c represents the scatter plots between the actual and predicted values for the best three models (i.e., ANN-GWO, ANN-ALO and ANN-GA) in the training phase based on the score analysis. In addition, Figure 9d–f illustrates the prediction performance for ANN-GWO, ANN-ALO and ANN-GA for the validation dataset (a portion of the training dataset). As can be seen, the developed ANN-GWO attained the most desired accuracy with R^2^ = 0.9720 and RMSE = 0.0230, followed by ANN-ALO (R^2^ = 0.9714 and RMSE = 0.0281) and ANN-GA (R^2^ = 0.9369 and RMSE = 0.0413).

From the analysis of the above hybrid models with the dimensionality-reduction method named PCA, we concluded that the ANN-GWO model again performed the best among all the models for the testing phase with the values of R^2^ = 0.9634 and MAE of 0.0230. The ANN-BBO, ANN-PSO and ANN ALO models provided satisfying results in terms of the statistical parameters. The RSME and WMAPE values for the above three models were near 0. The score analysis indicates that the ANN-DE and ANN-ABC models were again the underachieving models for testing. 

**Figure 10:**
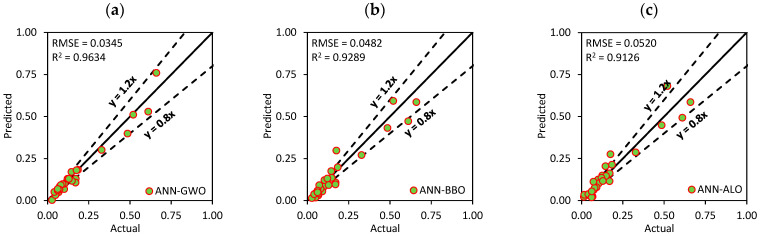
Scatter plots (**a**–**c**) between the actual and predicted values (for best three models—testing phase).

The performance of ANN-PSO significantly improved in the testing phase compared to the training phase with the value of R^2^ = 0.9274. In the training and testing phases, the RMSE values of the created models varied from 0.0291 to 0.0898 and 0.0345 to 0.1243, respectively. These results suggest that the generated models had a smaller error range, thereby, indicating a better level of accuracy. Figure 10 represents the scatter plots between the actual and predicted values for best three models (ANN-GWO, ANN-BBO and ANN-ALO) in the training phase.

In Table 8, the overall score analysis indicates that the most effective hybrid ANN model was ANN-GWO with the score of 128, followed by ANN-ALO, ANN-BBO and ANN GA with scores of 102, 98 and 70, respectively. ANN-ABC and ANN-DE gave the lowest overall scores of 22 and 26, respectively.

The visualisation of any results or dataset plays an important role in computational analysis. It helps to detect noise, pattern outliers and trends of the data, which make it easier to comprehend by the human brain. The graphical interpretations make it easy to identify the trends of the outcomes without going through the intimate details. By taking these things into the consideration, the following section presents the ‘accuracy matrix’ and ‘Taylor diagram’ to visualise the performance visualisation.

**Figure 11:**
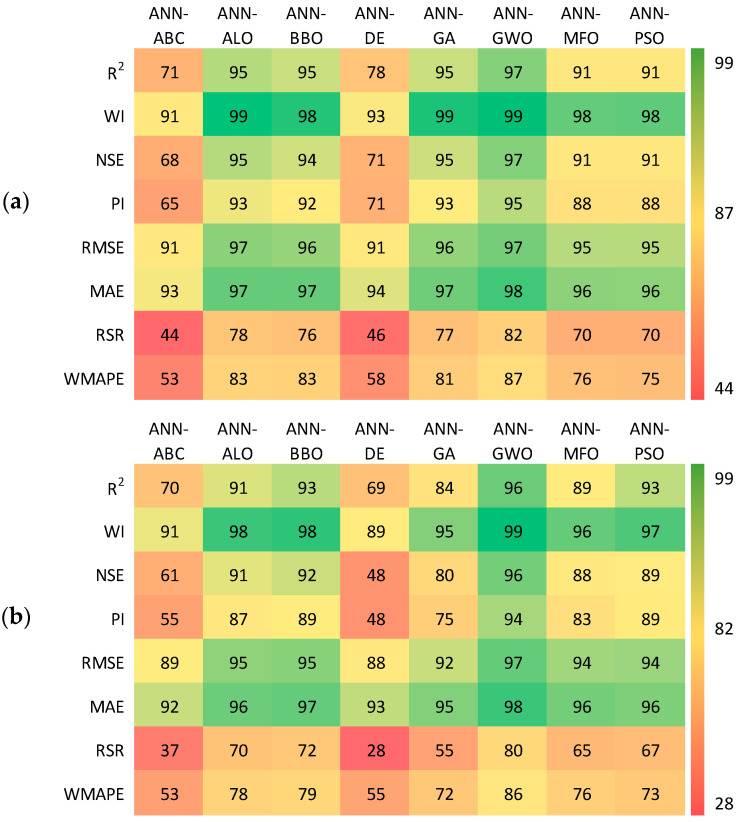
Accuracy matrices for the: (**a**) training phase and (**b**) testing phase.

For the accuracy matrix, a heat map is proposed to demonstrate the value of performance indices to visualise the model efficacy. In Figure 11, the accuracy matrix displays several statistical parameters to measure the performance of the prediction for the testing dataset of the model with PCA. The matrix provides the accuracy of models by comparison with the ideal values. For example, the ideal value of R^2^ is 1, and the value of R^2^ in testing phase for the ANN-GWO is 0.9667 in Table 6. Thus, it can be estimated that the models attained accuracy of 97% ((0.9667/1) × 100%) in terms of R^2^. 

On the other hand, the ideal value of MAE is 0, and the ANN-GWO has a value of MAE is 0.0191, which shows that the ANN-GWO attained 98% ((1−0.0191) × 100%) accuracy in terms of MAE. Hence, in the above manner, other parameters have been calculated as well. Through the study, it can be concluded that the ANN-GWO model outperformed the other models in both training and testing.

**Figure 12:**
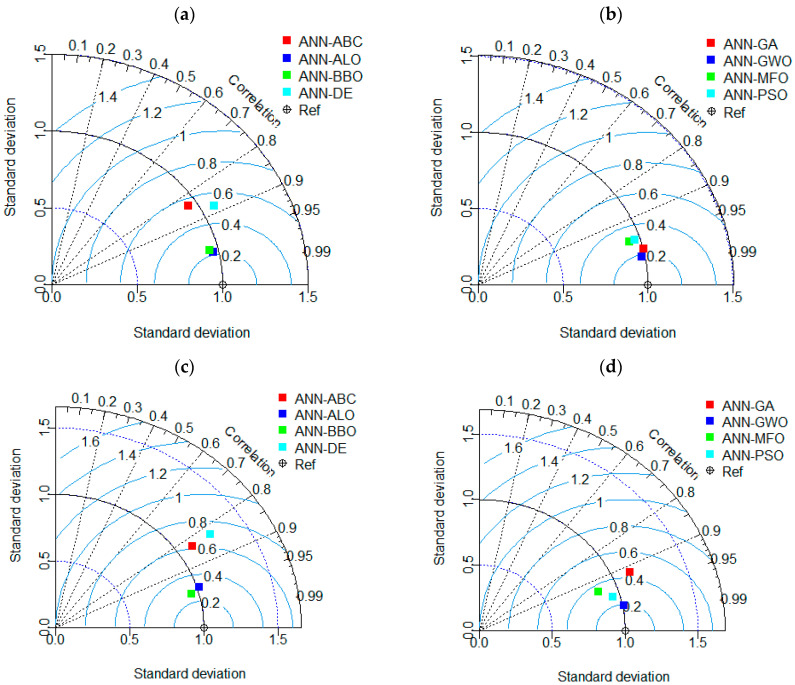
Taylor diagrams for the (**a**,**b**) training phase and (**c**,**d**) testing phase.

For the Taylor diagram, on the other hand, a 2D mathematical diagram is provided to represent the relation between the actual and predicted variables in terms of the RMSE, standard deviation and correlation coefficient. The status of all the models can be easily determined by the images provided in Figure 12. As can be observed, the Taylor diagram also provides the same result as seen in the accuracy matrix where ANN-GWO proved its supremacy over the other models in both phases.

### 5.3. Discussion of the Results

The section provides a summarised discussion of the proposed models for the prediction of ultimate bearing capacity of CFST columns. Based on the collected experimental results, it is clear that the proposed model defines the relationship between the input and output parameter accurately. The maximum prediction accuracy was achieved for ANN-GWO for the training stage with R^2^ = 0.9667, while the ANN-ABC underperformed among all the models in the training stage with R^2^ = 0.7094. 

The overall best-performing model was ANN-GWO followed by ANN-ALO with the value of R^2^ equal to 0.9527 in training and ANN-BBO with the value of R^2^ equal to 0.9289 in testing. Similar conclusions can be achieved from the rank table. Considering all the statistical parameters, the best-performing model was ANN-GWO with the rank of 128 followed by ANN-ALO, ANN-BBO and ANN GA with scores of 102, 98 and 70, respectively. The ANN-ABC model was found to be the most underperforming model among all the developed hybrid models. Similar performances were observed using the accuracy matrix and Taylor diagram.

## 6. Summary and Conclusions

It is pertinent to mention that an accurate and trustworthy prediction of the ultimate load-bearing capacity of CFST can save time and will make the process more economical. Based on the study, the following conclusions are made:

(a) In the current study, 149 experimental data of CFSTs under uniaxial load with eight input parameters were obtained from the literature survey. Some recently developed MOAs were employed with ANN.
(b) Among the models, ANN-GWO was the best-performing model in both phases followed by ANN-ALO (R^2^ = 0.9527, RSME = 0.0347, RSR = 0.2177) in the training stage and ANN-BBO (R^2^ = 0.9289, RSME = 0.0482, RSR = 0.2799) in testing. The ANN-ABC was the most underperforming model in the testing phase.
(c) A dimensionality-reduction method, PCA, was employed to increase the performance of the models. The experimental validation of the ANN-GWO using PCA demonstrated that it had higher prediction accuracy in both the training and testing stages. These results were significantly better than those obtained from the hybrid ANNs (ANN-ABC, ANN-ALO, ANN-BBO, ANN-DE, ANN-GA, ANN-GWO, ANN-MFO and ANN-PSO).
(d) Based on the experimental outcomes, the proposed ANN-GWO with PCA has the potential to assists structural engineers in estimating the P_u_ of CFSTs during the design phase of civil engineering projects. The proposed ANN-GWO can also be considered as a promising technique to handle real-life engineering problems, including the prediction of P_u_ of CFSTs. Some hybrid ANNs (i.e., ANN-ALO, ANN-BBO and ANN-GA) can be a good alternative to predict the P_u_ of CFSTs as they performed well for both phases.

## Figures and Tables

**Table 1:** Descriptive statistics of the employed dataset.

Particulars	*D*	*t*	*f_c_*	*E_c_*	*f_y_*	*E_s_*	*L*	*𝜉*	*P_u_*
Min.	60.00	0.86	18.03	17,810	186.00	177,000	180.00	0.05	312.00
Mean	164.38	3.71	65.60	34,907	339.85	201,767	485.07	0.86	2328
Max.	450.00	10.36	193.30	66,000	853.00	213,000	1760	3.22	13,776
Stnd. Error	5.17	0.17	3.82	1056	8.16	575.81	18.08	0.06	179.57
Stnd. Dev	63.09	2.08	46.58	12,890	99.57	7029	220.73	0.73	2192
Variance	3980	4.34	2170	166,153,299	9914	49,401,978	48,721	1	4,804,727
Kurtosis	5.18	1.90	0.80	−0.18	11.50	2.21	9.12	0.94	6.10
Skewness	1.79	1.40	1.24	0.83	2.75	−1.01	2.32	1.19	2.31

**Table 2:** Realizations of PCA.

Particulars	PC1	PC2	PC3	PC4	PC5	PC6	PC7	PC8
SDEV	1.5687	1.4209	1.3079	0.9342	0.8373	0.3400	0.2754	0.2105
POV	0.3076	0.2524	0.2138	0.1091	0.0876	0.0145	0.0095	0.0055
CPOV	0.3076	0.5600	0.7738	0.8829	0.9705	0.9850	0.9945	1.0000

**Table 3:** Rotations of PCs.

Parameters	PC1	PC2	PC3	PC4	PC5	PC6	PC7	PC8
D	0.0946	−0.6619	0.1731	0.0354	−0.0425	0.1215	0.7077	−0.0662
t	0.0462	−0.0442	0.6078	−0.5152	−0.3882	0.3771	−0.2604	−0.0190
*f* *_c_*	0.5987	0.1220	0.0210	−0.2380	0.0770	−0.3433	0.0421	−0.6663
E_C_	0.5993	0.1595	0.0687	−0.1635	0.0879	−0.1514	0.1597	0.7265
*f_y_*	0.0578	0.1034	0.5478	0.3167	0.7183	0.2447	−0.0665	−0.0723
E_S_	0.2425	0.1529	0.2783	0.7238	−0.5576	−0.0613	−0.0202	−0.0380
L	0.0929	−0.6706	0.0867	0.0939	0.0802	−0.4127	−0.5790	0.1152
𝜉	−0.4478	0.1894	0.4587	−0.1303	−0.0211	−0.6843	0.2530	0.0594

**Table 4:** The ideal values of different performance parameters.

Indices	R^2^	WI	NSE	PI	RMSE	MAE	MAPE	WMAPE
Ideal Value	1	1	1	2	0	0	0	0

**Table 5:** Model configuration.

Parameters	ANN-ABC	ANN-ALO	ANN-BBO	ANN-DE	ANN-GA	ANN-GWO	ANN-MFO	ANN-PSO
$N_{hn}$	10	10	10	10	10	10	10	10
$N_{hl}$	1	1	1	1	1	1	1	1
$N_{p}$	50	50	50	50	50	50	50	50
itr	500	500	500	500	500	500	500	500
$w_{\max}$	0.9	-	-	-	-	-	-	-
$w_{\min}$	0.4	-	-		-	-	-	-
$c_{1},c_{2}$	1, 2	-			-	-	-	-
$r_{1},r_{2}$	0–1	-	-	-	-	-	-	-
ub, lb	±1	±1	±1	±1	±1	±1	±1	±1
$N_{lp}$	101	101	101	101	101	101	101	101

**Table 6:** Model performance in the training phase.

Indices	Param.	ANN-ABC	ANN-ALO	ANN-BBO	ANN-DE	ANN-GA	ANN-GWO	ANN-MFO	ANN-PSO
R^2^	Value	0.7094	0.9527	0.9460	0.7761	0.9463	**0.9667**	0.9096	0.9095
	Score	1	7	5	2	6	**8**	4	3
WI	Value	0.9108	0.9876	0.9849	0.9300	0.9862	**0.9915**	0.9751	0.9759
	Score	1	7	5	2	6	**8**	3	4
NSE	Value	0.6830	0.9526	0.9434	0.7102	0.9453	**0.9667**	0.9090	0.9088
	Score	1	7	5	2	6	**8**	4	3
PI	Value	1.2959	1.8672	1.8497	1.4115	1.8504	**1.9020**	1.7641	1.7642
	Score	1	7	5	2	6	**8**	3	4
RMSE	Value	0.0898	0.0347	0.0380	0.0858	0.0373	**0.0291**	0.0481	0.0482
	Score	1	7	5	2	6	**8**	4	3
MAE	Value	0.0687	0.0252	0.0256	0.0610	0.0278	**0.0191**	0.0355	0.0359
	Score	1	7	6	2	5	**8**	4	3
RSR	Value	0.5630	0.2177	0.2380	0.5383	0.2339	**0.1824**	0.3016	0.3020
	Score	1	7	5	2	6	**8**	4	3
WMAPE	Value	0.4678	0.1685	0.1742	0.4151	0.1891	**0.1292**	0.2397	0.2451
	Score	1	7	6	2	5	**8**	4	3
Total score		8	56	42	16	46	64	30	26

**Table 7:** Model performance in the testing phase.

Indices	Param.	ANN-ABC	ANN-ALO	ANN-BBO	ANN-DE	ANN-GA	ANN-GWO	ANN-MFO	ANN-PSO
R^2^	Value	0.6954	0.9126	0.9289	0.6883	0.8419	**0.9634**	0.8869	0.9274
	Score	2	5	7	1	3	**8**	4	6
WI	Value	0.9051	0.9770	0.9792	0.8896	0.9535	**0.9900**	0.9647	0.9717
	Score	2	6	7	1	3	**8**	4	5
NSE	Value	0.6075	0.9086	0.9217	0.4790	0.7984	**0.9599**	0.8793	0.8935
	Score	2	6	7	1	3	**8**	4	5
PI	Value	1.0930	1.7364	1.7823	0.9505	1.5032	**1.8777**	1.6657	1.7708
	Score	2	5	7	1	3	**8**	4	6
RMSE	Value	0.1078	0.0520	0.0482	0.1243	0.0773	**0.0345**	0.0598	0.0562
	Score	2	6	7	1	3	**8**	4	5
MAE	Value	0.0773	0.0368	0.0348	0.0734	0.0463	**0.0230**	0.0401	0.0450
	Score	1	6	7	2	3	**8**	5	4
RSR	Value	0.6265	0.3023	0.2799	0.7218	0.4489	**0.2003**	0.3474	0.3264
	Score	2	6	7	1	3	**8**	4	5
WMAPE	Value	0.4706	0.2242	0.2120	0.4468	0.2815	**0.1399**	0.2438	0.2736
	Score	1	6	7	2	3	**8**	5	4
Total score		14	46	56	10	24	**64**	34	40

**Table 8:** Model performance in the training phase.

Phase	ANN-ABC	ANN-ALO	ANN-BBO	ANN-DE	ANN-GA	ANN-GWO	ANN-MFO	ANN-PSO
Training	8	56	42	16	46	**64**	30	26
Testing	14	46	56	10	24	**64**	34	40
Training + Testing	22	102	98	26	70	**128**	64	66

## Data Availability

The data used in this research have been properly cited and reported in the main text.
